# Coercive Control and Intimate Partner Violence: Relationship With Personality Disorder Severity and Pathological Narcissism

**DOI:** 10.1002/pmh.70038

**Published:** 2025-09-04

**Authors:** Nicholas J. S. Day, David Kealy, Marko Biberdzic, Ava Green, Georgia Denmeade, Brin F. S. Grenyer

**Affiliations:** ^1^ School of Psychology University of Wollongong Wollongong Australia; ^2^ Department of Psychiatry University of British Columbia Vancouver Canada; ^3^ Department of Psychology Bishop's University Sherbrooke Canada; ^4^ Department of Psychology City St George's, University of London London UK

**Keywords:** coercive control, grandiosity, intimate partner violence, pathological narcissism, personality functioning, vulnerability

## Abstract

Intimate partner violence (IPV) is a global health concern, with increasing efforts focused on detection and prevention. Coercive control has been identified as a ‘golden thread’ linking risk profiles and violence perpetration. Narcissistic pathology is often implicated in control and violence, but research linking narcissism with aggression and abuse has been inconsistent. Most research on narcissism focuses on symptomatology, whereas contemporary diagnostic frameworks emphasise a dimensional approach to personality disorder ‘severity’. No study has examined the association between pathological narcissism, violence and coercive control while accounting for overall personality pathology. Individuals in relationships with relatives high in narcissism (*N* = 135; 71% romantic partners, 22% family members; average relationship length = 20 years) completed informant measures of pathological narcissism and personality disorder severity, as well as self‐report measures of abuse and coercive control. Relatives were rated highly in both grandiose and vulnerable narcissism features, as well as displaying prominent impairments in personality functioning. Correlation analysis indicated dimensional personality disorder severity was significantly and moderately associated with both abuse and coercive control. Pathological narcissism was significantly associated with coercive control but not abuse. Specific narcissism subfactors (exploitativeness, grandiose fantasy and entitlement rage) showed positive, weak associations with either coercive control or abuse. Within the context of high narcissistic symptomatology, personality disorder severity may be a risk factor for coercive control and IPV. Clinical implications suggest the relevance of incorporating a focus on personality in psychological interventions targeted at reducing IPV and coercive control.

## 1 Introduction

Globally, more than a quarter (27%) of women have experienced physical or sexual abuse (or both) from an intimate partner (Sardinha et al. 2022), representing an international public health concern (World Health Organization 2021). Although intimate partner violence is disproportionately perpetrated by men against women, men are also victims of intimate partner violence, with prevalence rates ranging from 3.4% to 20.3% (physical violence), 7.3% to 37% (psychological violence) and 0.2% to 7% (sexual violence) (Kolbe and Buttner 2020). In an effort to improve the detection and prevention of domestic violence, there has been an increased focus over the last decade on investigating coercive control, described by some as the ‘golden thread’ linking risk profiles of dangerous patterns of behaviour and eventual domestic violence perpetration (Myhill and Hohl 2019). In their review, Hamberger et al. (2017) highlight that coercive control is itself not violence, but rather it is a controlling relational dynamic, a ‘condition of unfreedom’ (Stark 2007, 205), that is established and often maintained through the use of violence. To illustrate this, they use an evocative example of a woman who was systematically beaten by her husband while telling her to ‘never go against his will’ (p. 2)—as a consequence, years after any violence had ceased, she may still have intense emotional and fearful reactions to any sign of disapproval from him. In recognition of this, several countries and jurisdictions have begun to legislate coercive control as a criminal offense (Stark and Hester 2019), including England/Wales, Scotland and Australia.

### 1.1 Narcissism, Coercive Control and Violence

Pathological narcissism is a maladaptive personality constellation that contains grandiose (e.g., omnipotent fantasy and attention seeking) and vulnerable (e.g., hypersensitivity and negative affect) dimensions (Day et al. 2020b; Krizan and Herlache 2017; Pincus and Lukowitsky 2010). Irrespective of whether narcissistic features reach threshold for diagnosis (i.e., ‘narcissistic personality disorder’; American Psychiatric Association 2022), interpersonal dysfunction has been well established as a core concomitant (Biberdzic et al. 2023; Cheek et al. 2018; Kealy and Ogrodniczuk 2011; Ogrodniczuk et al. 2009). Such interpersonal patterns typically involve hostile and conflictual patterns of relating (Czarna et al. 2019; Day et al. 2022; Wright et al. 2017), but which may extend to coercive control (Felton 2024) and serious abuse and violence within intimate relationships (Green and Charles 2019). For instance, Day et al. (2021) report on the qualitative descriptions provided by a large informant sample that include themes of physical, verbal, emotional and sexual abuse, as well as coercive controlling behaviour (e.g., controlling finances) from individuals with reported narcissistic features. Exploring the link between narcissism, aggression and violence, a meta‐analysis by Kjaervik and Bushman (2021) found a weak but enduring significant association with narcissism, particularly when under provocation conditions. Similarly, a recent meta‐analysis and systematic review reported a significant but weak positive relationship between narcissism and intimate partner violence (Oliver et al. 2023). As such, given these consistent (but relatively low strength) associations, elucidating the specific components of narcissism that relate to aggression and/or violence within relationships has been an ongoing area of research. For example, specific associations identified within empirical literature include self‐control (Larson et al. 2014), early childhood experiences (Green et al. 2019; Green et al. 2024b; Green et al. 2020), self‐esteem (Lamarche and Seery 2019) and loneliness (Kealy et al. 2022). Although these studies have been critical in understanding specific manifestations of narcissistic pathology, there is a need to unify these disparate foci within an integrated framework.

### 1.2 Personality Disorder ‘Severity’

The publication of the DSM‐5 Alternative Model of Personality Disorder (American Psychiatric Association 2013) reflected a major metatheoretical and methodological shift towards conceptualising personality disorder along a spectrum (Hopwood et al. 2018), with the ICD‐11 recently publishing a nearly identical dimensional model within its system (World Health Organization 2024). In these systems, personality disorder is assessed according to core features of ‘Self’ (e.g., identity, self‐direction) and ‘Interpersonal’ (e.g., empathy and intimacy) functioning, indexed according to a dimension of severity of impairment (e.g., mild, moderate and severe; for more detailed explanation, see Bach and First 2018). Empirical, theoretical and clinical support for the dimensional models has been slowly accumulating in the last decade (Bach and Tracy 2022; Sharp et al. 2015; Weekers et al. 2024b; Wright et al. 2016); however, challenges remain in demonstrating superiority and acceptability (Bach et al. 2022; Sharp and Miller 2022; Weekers et al. 2024a). Regarding narcissism specifically, recent explorations have suggested the utility of the dimensional model in capturing core features of narcissistic personality functioning across diverse (i.e., grandiose and vulnerable) expressions (Day et al. 2024; Green et al. 2024a), something that categorical models have struggled to achieve (Levy et al. 2013).

However, although there is novelty in the fact that these dimensional models have been adopted by mainstream classification and diagnostic systems, the concept of personality organised according to spectra is itself not new (e.g., Psychodynamic Diagnostic Manual; Kernberg 1967; Lingiardi and McWilliams 2017; PDM Task Force 2006). Key theoreticians in this tradition have historically proposed there to be a particularly severe and extreme form of narcissistic functioning (termed ‘malignant’; Kernberg 2008). Although this more severe manifestation of pathological narcissism has typically been discussed primarily in clinical and theoretical literature (e.g., Kernberg 2007; Kernberg 2020), it has demonstrated some empirical support (Russ et al. 2008), particularly in recent years (Cain et al. 2024; Lenzenweger et al. 2018). As such, as it relates to coercive control and violence perpetration, severe dysfunction in pathological narcissism may involve rage or dominance towards others, coupled with a loss of empathy for others' experience of such behavior. However, associations between narcissism, personality functioning, coercive control and intimate partner violence have not been systematically explored within the literature to date and are the primary aim of the current research.

### 1.3 The Current Study

The current study has two main related aims. First, we aim to explore participants' experiences of coercive control and abuse within the relationship with their relative with reported high narcissistic features. Second, we aim to index perceptions of pathological narcissism and severity of impairment in personality functioning as rated by close informants (i.e., partners and family members) and to explore associations between these personality‐related variables and the endorsement of coercive control and abuse. There are well‐documented challenges in empirically examining narcissism, such as reliance on self‐report methods and/or utilising predominately non/sub‐clinical samples (Miller and Campbell 2010). Although not itself being free of methodological limitations, utilising an informant sample has been argued to be a potentially valuable approach that avoids some of these common issues (Lukowitsky and Pincus 2013; Oltmanns et al. 2018; Pincus and Lukowitsky 2010), as well as providing a unique and important perspective in itself. For this research, individuals completing informant and self‐report measures will be referred to as ‘participants’, whereas the individuals with pathological narcissism (typically romantic partners or family members) will be referred to as ‘relatives’.

## 2 Method

### 2.1 Recruitment

Participants were recruited through invitations posted on various mental health websites and social media groups that provide information and support to individuals who have a relationship with someone with narcissistic features (e.g., ‘Narcissistic Family Support Group’ Facebook page). This strategy for data collection has been found to be effective and reliable (Day et al. 2020a; Miller et al. 2017). Inclusion criteria were that participants needed to identify as having a close personal relationship with someone with pathological narcissism and be able to read and understand English. Participants provided written informed consent to participate following institutional review board approval.

### 2.2 Participants

A total of 207 participants consented to participate in this study. To support participant well‐being, it was instructed that participants could skip specific items directly inquiring about the nature of any abuse experienced, and this would not impact their inclusion in the study. Only 7% of participants chose to skip any questions detailing specific instances of abuse; however, these participants were still retained for overall analysis. The remaining questions (i.e., relating to demographics, narcissism and personality severity) were required for inclusion, and participants who did not complete these measures were removed (*n* = 72). The remaining 135 participants formed the sample reported in this study. Table 1 outlines the demographics information of the participants and their relatives included in this study.

**TABLE 1:** Demographics for participants (partners and family) and their relatives (people high in pathological narcissism) (*N* = 135).

	Participants (*N* = 135)	Relative (*N* = 135)
Mean age in years (SD, range)	47.3 (11.3, 20–71)	53.6 (13.9, 30–91)
Gender		
Male	11.1%	63%
Female	86.7%	27.4%
Not specified	2.2%	9.6%
Employment		
Full time	48.9%	49.6%
Part time	11.9%	7.4%
Unemployed	15.6%	9.6%
Other (e.g., studying and disability)	21.5%	32.6%
Relationship type		
Partner/spouse	71.1%	
Mother	16.3%	
Father	1.5%	
Sibling	3.7%	
Child	0.7%	
Other (e.g., extended family, roommate and colleague)	6.7%	
Relationship length in years (SD, range)	20.3 (16.6, 1–91)	
Still in contact?		
Yes	51.1%	
No	48.9%	
Frequency of contact		
Daily	46.9%	
Weekly	18.8%	
Monthly	7.8%	
Yearly	3.1%	
Infrequently/inconsistently	23.4%	

### 2.3 Measures

#### 2.3.1 Interpersonal Violence and Abuse

The revised short form version of the Composite Abuse Scale (CASR‐SF; Ford‐Gilboe et al. 2016) was utilised in this study to measure participants' experience of physical, sexual and psychological abuse from their relative with features of narcissism. The measure consists of 15 items that directly ask about experiences of abuse (e.g., ‘hit or tried to hit me with a fist or object, kicked or bit me’) and are scored on a 6‐point Likert scale from 0 = *never* to 5 = *daily*. As outlined by Hamberger et al. (2017), there is a need for research to delineate between interpersonal violence/abuse and coercive controlling behaviour (and particularly between psychological abuse and coercive control, as psychological abuse may or may not involve controlling tactics). As such, this measure is a direct examination of abusive behaviour specifically, and we utilised the 12 items that demonstrated a loading onto either psychological, physical or sexual abuse, respectively (Ford‐Gilboe et al. 2016). Internal consistency of the CASR‐SF was good (α = 0.85).

#### 2.3.2 Coercive Control

The Controlling Behaviours Scale (CBS; Graham‐Kevan and Archer 2005) is a 24‐item measure designed to assess the behaviours perpetrated in the context of abuse or coercive control scored on a 5‐point Likert scale from 0 (*never*) to 4 (*always*). The CBS captures five domains of controlling behaviours: economic control, threatening control, intimidating control, emotional control and isolating control. For example, ‘check up on other's movements’ falls within the ‘isolating control’ domain. As prior, following recommendations by Hamberger et al. (2017), this measure is a direct examination of coercive control (as separate from violence) within these relationships regarding intentional acts done against participants in an effort to restrict their freedom and exercise power of the perpetrator. Internal consistency for the CBS was excellent (α = 0.93).

#### 2.3.3 Pathological Narcissism

The Brief Pathological Narcissism Inventory (B‐PNI; Pincus et al. 2009; Schoenleber et al. 2015) was used to measure pathological narcissism in the relatives of participants. The PNI is a measure explicitly geared towards non‐adaptive, pathological expressions of both grandiose and vulnerable narcissism (Krizan and Herlache 2017). The PNI is a widely used measure of narcissism with demonstrated validity and reliability (Kaufman et al. 2018; Weiss et al. 2020). The measure has also been used in an informant version (Lukowitsky and Pincus 2013), and in this study, items are altered from self‐report (e.g., ‘I’) to informant report (e.g., ‘my relative’) wording as consistent with prior research (Day et al. 2020b). The B‐PNI contains 28 items, scored on a 6‐point scale ranging from 0 (*not at all like my relative*) to 5 (*very much like my relative*). The B‐PNI contains six subscales, which load onto grandiosity (‘exploitativeness’, ‘self‐sacrificing self‐enhancement’, ‘grandiose fantasy’) and vulnerability (‘contingent self‐esteem’, ‘hiding the self’, ‘devaluing’ and ‘entitlement rage’). Internal consistency was good for the whole measure (α = 0.85), the vulnerable subscale (α = 0.81), and was acceptable for the grandiose subscale (α = 0.72).

#### 2.3.4 Personality Functioning

The Personality Disorder Severity Scale (PDS‐ICD‐11; Bach et al. 2021) was utilised to measure personality disorder severity as per participant informant ratings of their relative. The PDS‐ICD‐11 captures core elements of personality functioning: self‐functioning, interpersonal functioning, emotional, cognitive and behavioural manifestations, harm to self and others and global impairment. This measure has been shown to be a valid and reliable measure of personality disorder severity in clinical samples (Brown and Sellbom 2023). Items 1–10 covering self, interpersonal, behavioural and emotional functioning are rated on a 5‐point scale (i.e., 2–1–0–1–2), with the centre point (‘0’) representing healthy functioning and outer points (‘2’) representing impairment. Whereas the remaining items covering reality testing, harm to self and others and global impairment are scored on a 4‐point scale (i.e., 0–1–2–3) where higher scores indicate greater impairment. Similar to prior research (e.g., Day et al. 2024), the PDS‐ICD‐11 was scored in two different ways in the current research. First, the items are simply summed to provide an overall score of impairment and compared against published comparisons to indicate personality disorder caseness (Bach et al. 2021). Second, as items 1–10 exist on a bipolar dimension, with each extreme reflecting the opposite manifestation (e.g., for the ‘identity’ aspect, extreme scores capture either ‘absent’ or ‘rigid’ identity, respectively), the scale was re‐coded to a −2 to +2 scale to meaningfully capture differences in expression of personality impairment. Internal consistency was acceptable (α = 0.69).

### 2.4 Data Analysis

Associations between variables were explored via correlation analysis using SPSS (v28). Missing data of the included sample were less than 1%, which is deemed tolerable (Dong and Peng 2013) and does not require data transformation. The Shapiro–Wilk test of normality indicated that scores on the CASR‐SF (abuse) and CBS (coercive control) were normally distributed (*p* > 0.05), and this was confirmed by inspection of histograms. Scores on the B‐PNI (pathological narcissism) and PDS‐ICD‐11 (personality functioning) indicated a violation of the normality assumption (*p* < 0.05); however, whereas the B‐PNI histogram showed a clear negative skew, the PDS‐ICD‐11 appeared to be only slightly skewed. This is perhaps not surprising, as we were seeking a sample of individuals with suspected narcissistic pathology; it might be expected that these scores trended towards the higher end. Parametric (Pearson) correlation analysis was nonetheless utilised despite these violations: (1) The key variables under examination (i.e., abuse and coercive control) were normally distributed. (2) The sample size was robust to tolerate some deviation in normality (Bishara and Hittner 2015). (3) Comparisons between parametric and non‐parametric tests indicated the same pattern of results.

## 3 Results

Self‐reported instances of experiencing abuse and coercive control are presented in Table 2, along with informant‐rated measures of pathological narcissism and personality disorder severity. Participants reported experiencing interpersonal abuse from their relative, most frequently indicating psychological abuse (e.g., denigration and name calling) with the majority of participants reporting this to occur regularly. Although experiencing physical (e.g., being hit, kicked and bit) and sexual abuse (e.g., forced sex) was less common, approximately a quarter of participants reported this to occur on more than one occasion, with a smaller proportion of participants indicating this occurred frequently in their relationships. Interestingly, comparisons of demographic factors indicated no significant variation in reported instances of abuse as a function of relationship type (i.e., romantic or familial), relationship status (i.e., current or former) or frequency of contact (i.e., daily, weekly, monthly and yearly).

**TABLE 2:** Clinical characteristics—relatives reported personality features and participants reported experience of abuse and coercive control.

Measure	Subfactor(s)	M (SD)/%
Pathological Narcissism Inventory		3.6 (0.7)
	Grandiosity	3.8 (0.7)
	Exploitativeness	4.2 (0.7)
	Grandiose fantasy	2.5 (1.2)
	Self‐sacrificing self‐enhancement	3.5 (1.2)
	Vulnerability	3.5 (0.8)
	Contingent self‐esteem	3.4 (1.1)
	Hiding the self	3.8 (1.2)
	Devaluing	3.5 (1.1)
	Entitlement rage	4.5 (0.8)
Personality Disorder Severity Scale		22.2 (4.3)
	Indicate caseness for personality disorder?^a^	
	Yes	84%
	No	16%
Composite Abuse Scale		1.9 (0.9)
	Psychological abuse	2.8 (1.1)
	Never/once	21%
	A few times/monthly	39%
	Weekly/daily	39%
	Sexual abuse	1.3 (1.6)
	Never/once	61%
	A few times/monthly	24%
	Weekly/daily	15%
	Physical abuse	1 (1)
	Never/once	68%
	A few times/monthly	26%
	Weekly/daily	9%
Controlling Behaviours Scale		2.3 (0.8)
	Economic	2.5 (1.1)
	Threatening	1.5 (1)
	Intimidating	2 (1)
	Emotional	2.7 (1)
	Isolating	2.6 (1.2)

^a^ Caseness for personality disorder indicated by a summed score of 17.5 or higher (Bach et al. 2021).

Participant's average informant narcissism score is higher than published self‐report population means (e.g., M = 2.38, SD = 0.97; Schoenleber et al. 2015) but is consistent with prior research utilising an informant sample (e.g., M = 3.7, SD = 0.8; Day et al. 2020a; Day et al. 2022). Informant ratings also suggest relatives to display core features of personality disorder, with the majority meeting the threshold for caseness of personality disorder as per suggested cut‐scores (Bach et al. 2021).

### 3.1 Pathological Narcissism

There was a significant positive relationship with informant narcissism total scores and reported experiences of coercive control (*r* = 0.18, *p* = 0.04) but not abuse (*r* = 0.09, *p* = 0.34). Averaged subfactors relating to narcissistic ‘grandiosity’ were also related to reported experiences of coercive control (*r* = 0.19, *p =* 0.03), but again not abuse (*r* = 0.16, *p* = 0.08). There were, however, significant associations with specific grandiosity subfactors (i.e., exploitativeness and grandiose fantasy) and reported experiences of abuse and coercive control; these are displayed in Table 3.

**TABLE 3:** Associations between B‐PNI ‘Grandiosity’ subfactors, abuse and coercive control.

	Exploitativeness	Self‐sacrificing self‐enhancement	Grandiose fantasy
Abuse (CASR‐SF)	0.19*	−0.01	0.17
Psychological	0.12*	0.07	0.14
Physical	0.17	−0.08	0.06
Sexual	0.04	−0.07	0.22*
Coercive control (CBS)	0.18*	0.06	0.18*
Economic	0.16	−0.05	0.18
Threatening	0.19*	0.06	0.09
Intimidating	0.19*	0.09	0.18*
Emotional	0.09	0.08	0.13
Isolating	0.12	0.04	0.15

* Significant at *p* < 0.05.

** Significant at *p* < 0.001.

Averaged subfactors of narcissistic ‘vulnerability’ were not significantly associated with coercive control (*r* = 0.13, *p* = 0.14) or abuse (*r =* 0.02, *p* = 0.82). There were however again significant associations with specific vulnerability subfactors (i.e., entitlement rage and negative association with contingent self‐esteem) and reported experiences of abuse and coercive control; these are displayed in Table 4.

**TABLE 4:** Associations between B‐PNI ‘Vulnerability’ subfactors, abuse and coercive control.

	Contingent self‐esteem	Hiding self	Devaluing	Entitlement rage
Abuse (CASR‐SF)	−0.09	−0.05	0.08	0.19*
Psychological	−0.01	−0.01	0.11	0.22*
Physical	−0.20*	−0.09	−0.07	0.04
Sexual	−0.06	−0.05	0.13	0.14
Coercive control (CBS)	0.08	−0.03	0.11	0.31**
Economic	0.15	−0.06	0.04	0.24**
Threatening	0.01	−0.003	0.13	0.13
Intimidating	−0.001	0.000	0.09	0.35**
Emotional	0.01	−0.02	0.11	0.26**
Isolating	0.12	−0.04	0.07	0.28**

* Significant at *p* < 0.05.

** Significant at *p* < 0.001.

Averaged informant ratings of narcissistic grandiosity and narcissistic vulnerability were also significantly and positively associated with each other, *r* = 0.49, *p* < 0.001.

### 3.2 Personality Disorder Severity

**FIGURE 1:**
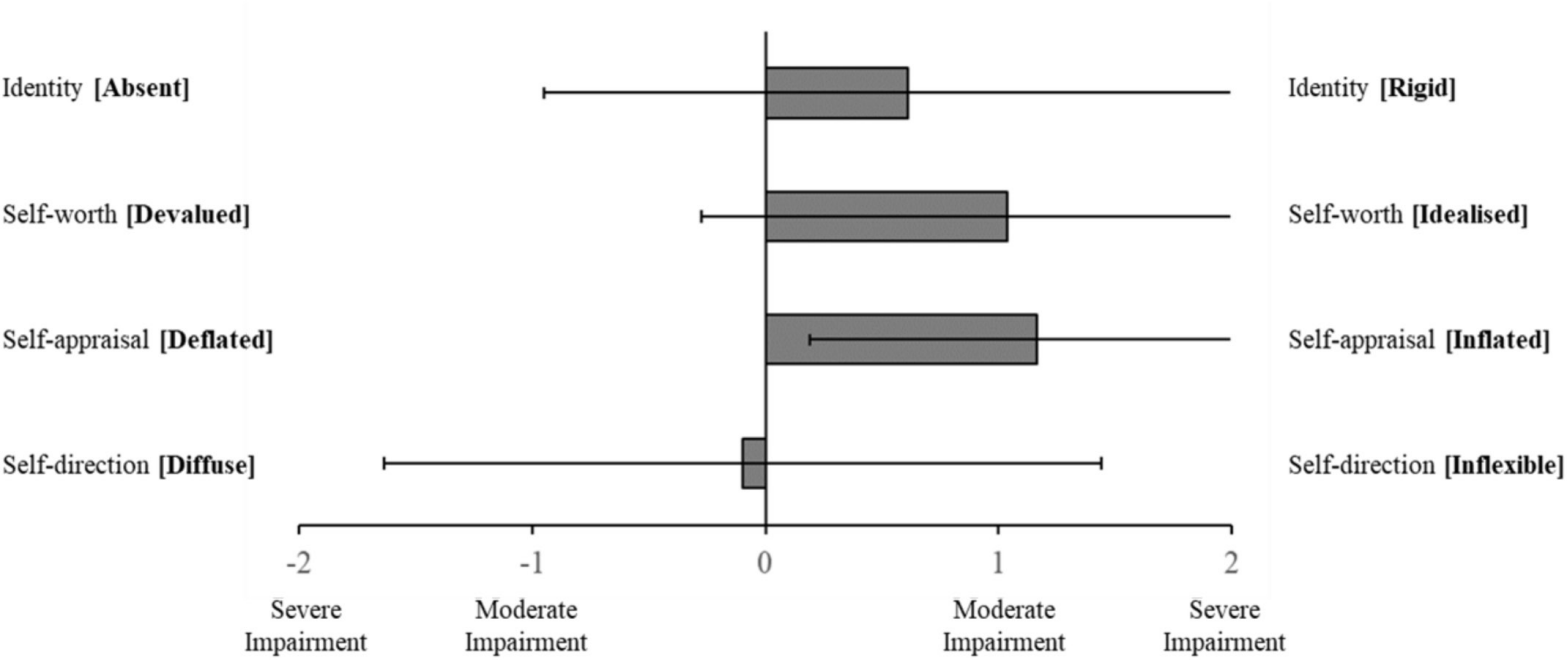
Mean informant ratings of impairment in relatives' aspects of self‐functioning. *Note:* Error bars indicate standard deviation.

Mean informant ratings of relatives' impairment in aspects of self‐functioning are visualised in Figure 1. These means reveal a trend towards more rigid identity (*M* = 0.61, SD = 1.56), idealised self‐worth (*M* = 1.04, SD = 1.31) and inflated self‐appraisal (*M* = 1.16, SD = 0.97). Ratings of self‐direction were less consistent, fluctuating between extremes of either diffusion or inflexibility (*M* = −0.10, SD = 1.54).

**FIGURE 2:**
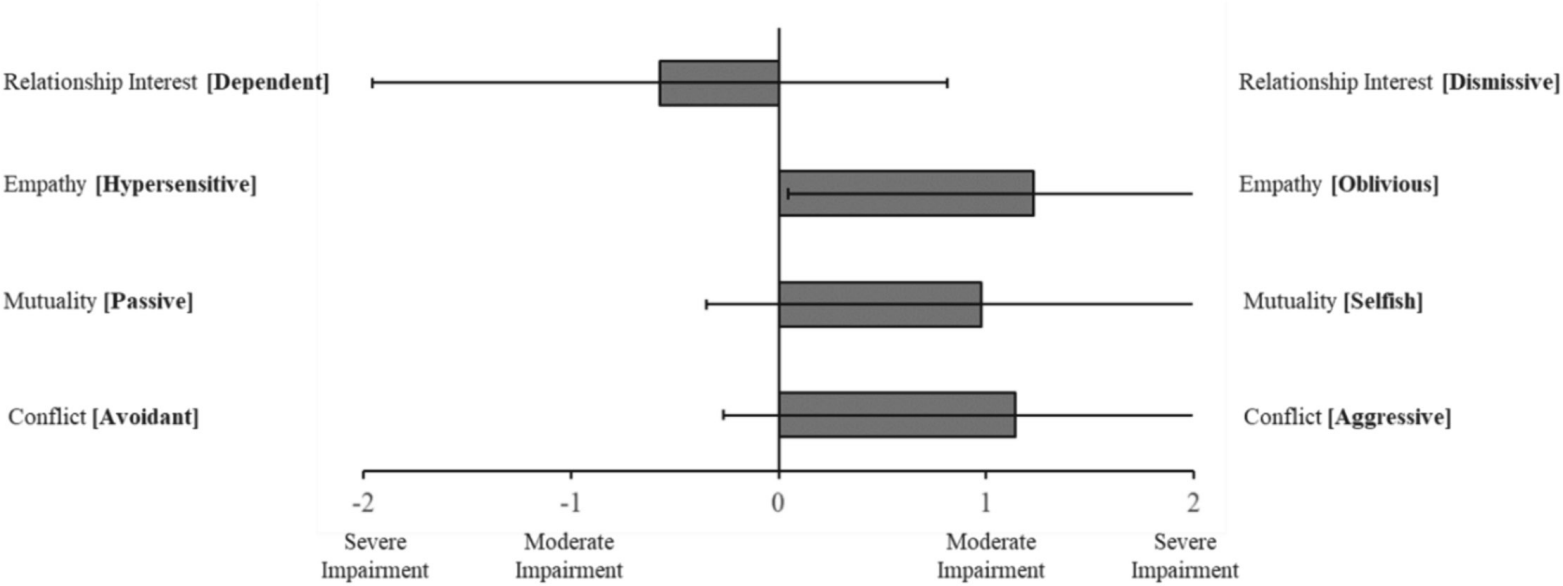
Mean informant ratings of impairment in relatives' aspects of interpersonal functioning. *Note:* Error bars indicate standard deviation.

Figure 2 displays mean informant ratings of relatives' impairment in aspects of interpersonal functioning. These means showed trends towards dependency in relationships (*M* = −0.057, SD = 1.39), oblivious empathic attunement (*M* = 1.23, SD = 1.18), low mutuality (*M* = 0.98, SD = 1.32) and high interpersonal conflict (*M* = 1.14, SD = 1.40).

**FIGURE 3:**
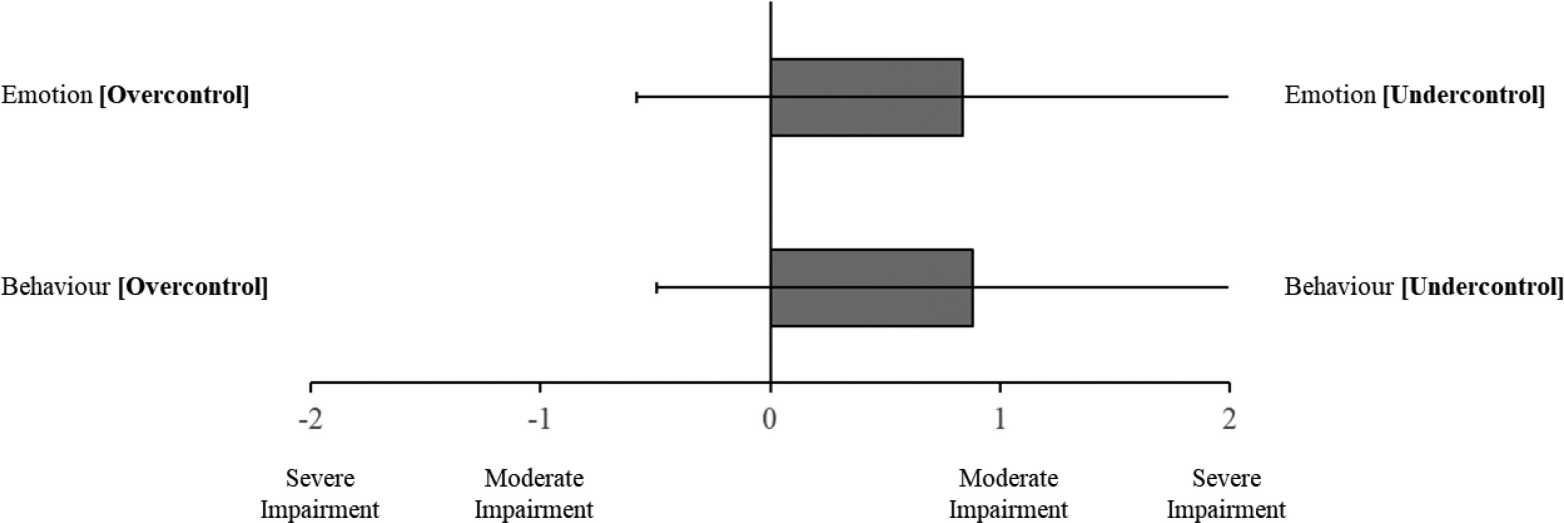
Mean informant ratings of impairment in relatives' emotional and behavioural control. *Note:* Error bars indicate standard deviation.

Mean ratings of relatives' impairment in emotional and behavioural control are depicted in Figure 3. These ratings revealed trends towards both emotional and behavioural under control (*M* = 0.84, SD = 1.42; *M* = 0.88, SD = 1.38, respectively).

**FIGURE 4:**
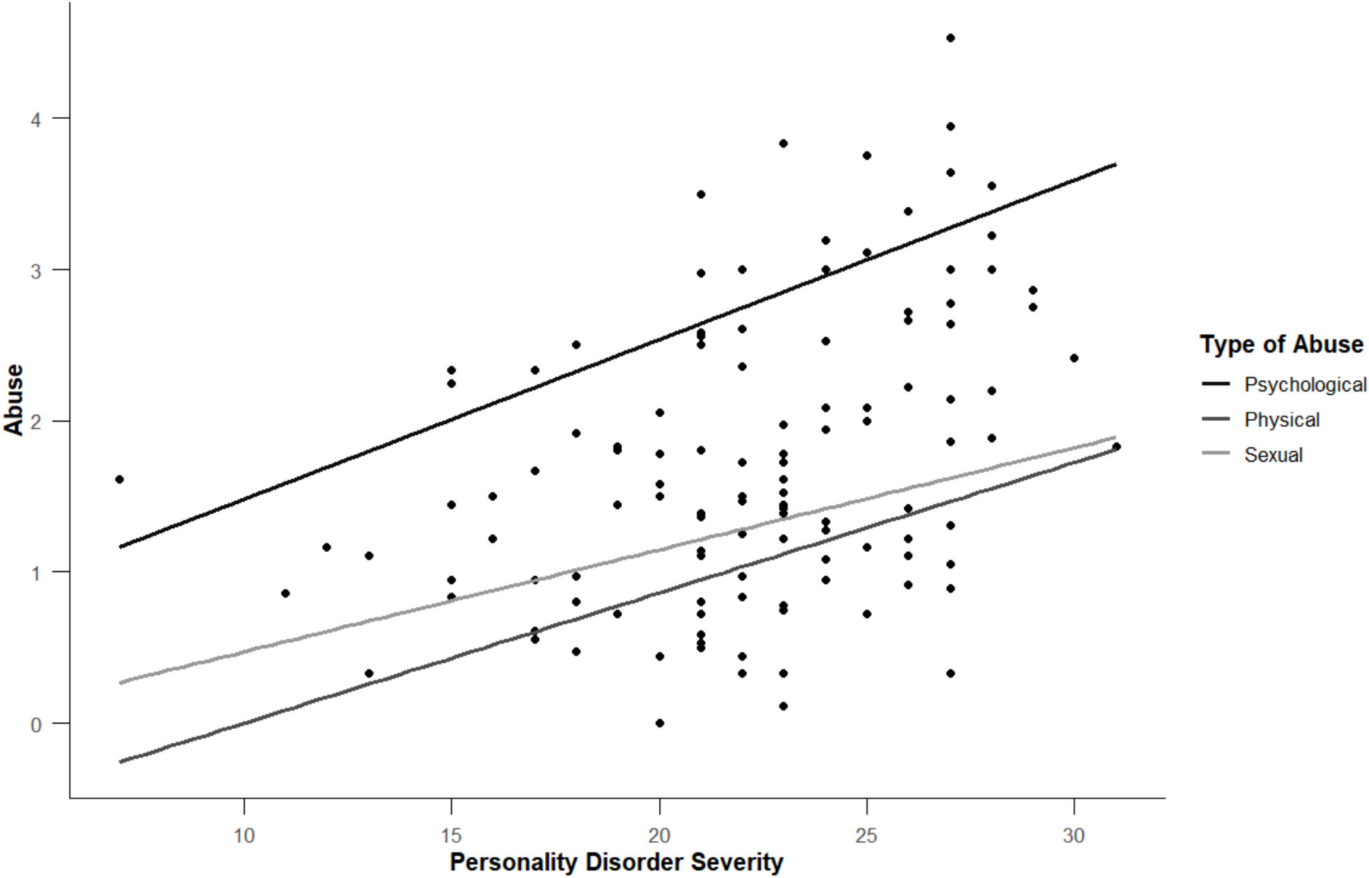
Association between overall personality disorder severity ratings and reported experiences of psychological, physical and sexual abuse.

**FIGURE 5:**
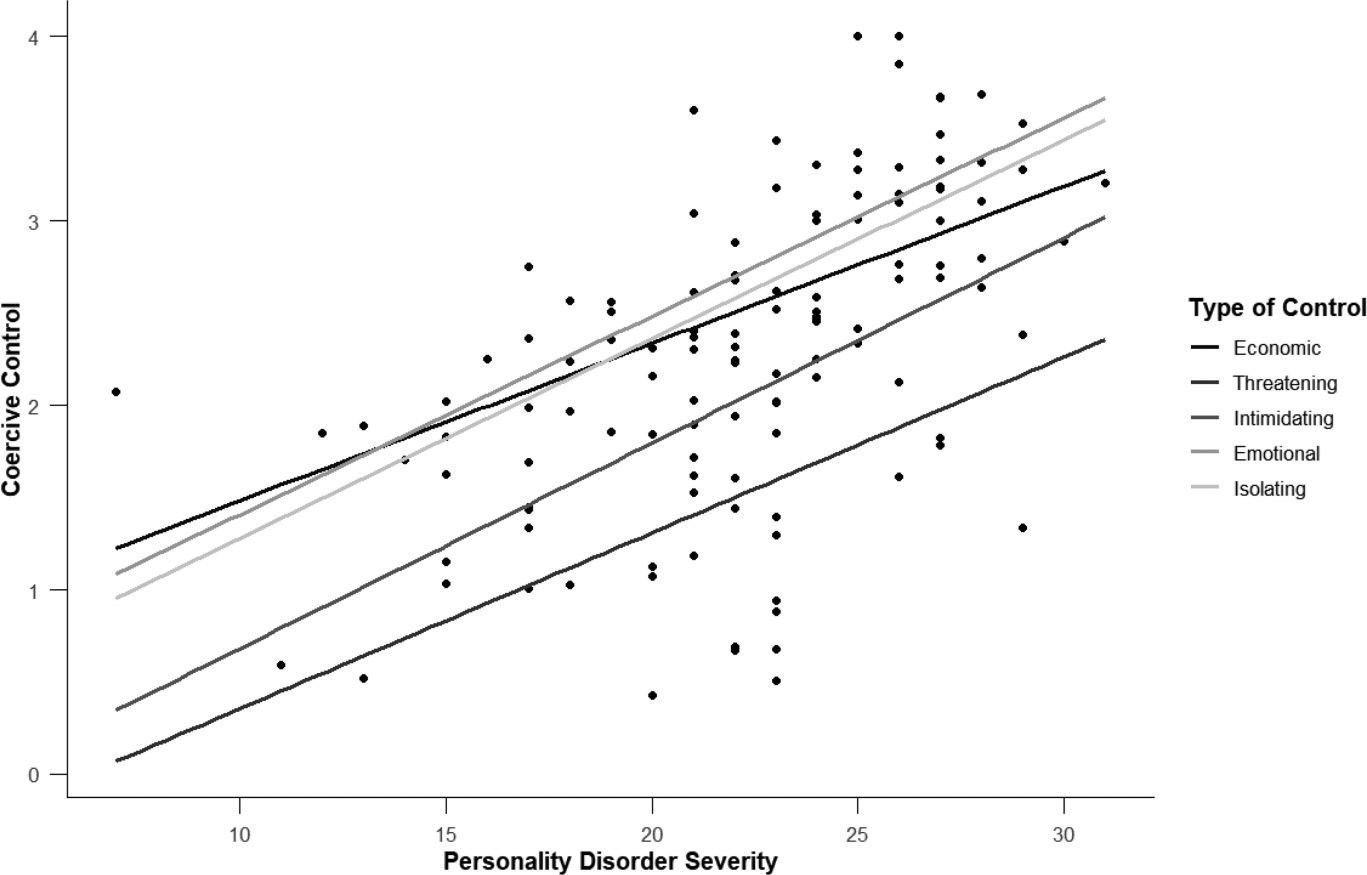
Association between overall personality disorder severity ratings and reported aspects of coercive control.

There was a significant, positive relationship between overall personality disorder severity and reported experiences of both abuse (*r* = 0.43, *p* < 0.001) and coercive control (*r* = 0.53, *p* < 0.001). These relationships are depicted in Figures 4 and 5. Table 5 displays the associations between each specific subfactor of personality functioning and experiences of abuse and coercive control.

**TABLE 5:** Associations between PDS‐ICD‐11 aspects of personality functioning, abuse and coercive control.

	Self‐functioning	Interpersonal functioning	Emotional‐behavioural control	Reality testing	Harm to self	Harm to others	Global impairment
Abuse (CASR‐SF)	0.25**	0.32*	0.22*	0.19*	0.18	0.37**	0.23*
Psychological	0.21*	0.32**	0.19*	0.22*	0.15	0.32**	0.26**
Physical	0.25*	0.24**	0.21*	0.16	0.14	0.28**	0.14
Sexual	0.12	0.14	0.07	0.001	0.12	0.26**	0.05
Coercive control (CBS)	0.34**	0.39**	0.32**	0.26**	0.13	0.43**	0.29**
Economic	0.21*	0.25**	0.19*	0.16	0.13	0.28**	0.25**
Threatening	0.32**	0.33**	0.32**	0.17	0.11	0.29**	0.19*
Intimidating	0.32**	0.38**	0.29**	0.22*	0.11	0.49**	0.32**
Emotional	0.29**	0.34**	0.31**	0.29**	0.01	0.42**	0.30**
Isolating	0.27**	0.33**	0.22*	0.21*	0.18*	0.23**	0.14

* Significant at *p* < 0.05.

** Significant at *p* < 0.001.

There was also a very strong, positive association between average self‐reported experiences of abuse and coercive control, *r* = 0.79, *p* < 0.001.

## 4 Discussion

This study sought to examine coercive control and violence within intimate relationships, exploring associations with pathological narcissism and personality functioning. Our first main finding was that total pathological narcissism scores were significantly but weakly associated with coercive control and not associated with abuse. This is largely consistent with research that has similarly reported only weak associations between narcissism, aggression and violence (Kjaervik and Bushman 2021; Oliver et al. 2023). In explaining these unexpected results, authors have pointed to potential social desirability bias for participants to underreport both their narcissism features and violence perpetration via self‐report measures. We mitigate this issue by using an informant sample, and yet, narcissism scores still did not display robust associations. However, despite the identified weak association, it is not necessarily accurate to conclude that narcissism is not implicated in coercive control and violence perpetration as, on average, informants rated quite high levels of pathological narcissism in their relatives. That is, this sample was high in narcissistic pathology; however, it may be the case that simply ‘more’ narcissism symptomatology is not associated with higher instances of coercive control or violence. In other words, pathological narcissism may be a necessary but not sufficient condition for the expression of control and violence within these relationships. Rather, for individuals with a baseline of prominent narcissistic pathology, appreciation of the level of personality functioning may be a useful adjunct as it captures a broader range of impairment such as aggressivity, self‐regulation, externalising tendencies and moral functioning (American Psychiatric Association 2022).

As such, our second main finding surrounds the significant, positive association reported between ratings of impaired personality functioning and reported experiences of coercive control and violence. Although the strength of our correlations is moderate (Mukaka 2012), this is not insubstantial. For instance, a one‐point difference on the CASR‐SF may equate to the difference between experiencing physical abuse ‘monthly’ and ‘weekly’—in other words, even moderate increases may translate to a substantial impact in terms of lived experience reality. Regarding specific impairments of personality functioning, it is interesting to account for the ‘direction’ of impairment as visualised in Figures 1, 2, 3, which provide some insight into the character of the relatives being reported on and particularly as it relates to the interplay of grandiosity and vulnerability (Caligor and Stern 2020). That is, although relatives predominately had rigid, idealised and inflated self‐views, there was also some endorsement of identity diffusion, as well as polarised goal‐setting capacity (aimlessness vs. unrealistic standards). Similarly, although relatives were rated as relationally unempathetic, selfish and conflictual, they were simultaneously rated as highly interpersonally needy or dependent. This comingling of contradictory haughty and fragile elements of personality functioning appears to replicate the observation of grandiosity and vulnerability being two sides of the same coin in pathological narcissism (Levy 2012). Fundamentally, however, these results support dimensional models of personality disorder (e.g., DSM‐5‐AMPD, ICD‐11 and PDM‐2), which account for a degree of impairment in core domains of self‐interpersonal functioning (American Psychiatric Association 2022; Lingiardi and McWilliams 2017; World Health Organization 2024). Whereas previous studies have identified the link between personality disorder and intimate partner violence (Collison and Lynam 2021; Rodríguez Hernández et al. 2024), most focus exclusively on BPD and fewer still specifically include an indication of severity of functioning beyond symptomatology (Munro and Sellbom 2020, as an exception). To our knowledge, no studies have focused on narcissistic pathology and accounted for severity of impairment in personality functioning when exploring intimate partner violence.

Nonetheless, our results indicate that some elements of narcissistic functioning may be related to coercive control and violence, specifically exploitativeness, grandiose fantasy and entitlement rage. This is perhaps not surprising, as exploitativeness definitionally involves interpersonal manipulation (Pincus 2013), which shares conceptual associations with coercive control (Hamberger et al. 2017). Similarly, Krizan and Johar (2015) highlight the link between narcissistic vulnerability and ‘narcissistic rage’, as threats to self‐esteem are felt as catastrophic due to underlying identity instability, motivating explosive anger and hostility as attempted self‐regulatory efforts. Grandiose fantasy, on the other hand, is likely implicated due to its role as an underlying intrapsychic motivational system that gives rise to such challenging interpersonal behaviours (Kernberg 2008). Associations between individual narcissism subfactors and specific instances of coercive control or abuse are also of interest. For instance, the significant association between grandiose fantasy and sexual abuse perpetration may indicate how indulging in fantasies of superiority may contribute to sexually coercive or entitled behaviour. Indeed, Lamarche and Seery (2019) report that individuals high in narcissism (but low in self‐esteem) were more likely to be more permissive of sexual coercion. Similarly, the association between grandiose fantasy and threatening and intimidating control may reflect efforts to force others into submission as a way of managing the paranoia that can accompany grandiose states, as part of the broader effort to maintain a sense of ‘omnipotent control’ in pathological narcissism (Kernberg 1985, 2008). Entitlement rage appears particularly salient, showing associations with almost all forms of coercive control indices (i.e., economic, intimidating, emotional and isolating). This reactive anger and hostility has been similarly documented in other studies on pathological narcissism (Czarna et al. 2019) due to the underlying hypersensitivity in pathological narcissism related to perceived threats to entitlement, agency and control (Ronningstam 2017). Finally, it is interesting the observed significant negative association between contingent self‐esteem and physical abuse. It may be that individuals high in this trait may avoid overt, physically aggressive behaviours that could damage their social image or jeopardise sources of affirmation. Alternatively, it may suggest that individuals low in this feature (e.g., more antisocial, less interpersonally motivated individuals) may be more prone to impulsive or dysregulated expressions of aggression, including physical violence.

## 5 Clinical Implications

There are a number of practice implications that stem from the current study. First, although research demonstrates that treatments for people who use violence and engage in coercive control are generally effective (Karakurt et al. 2019; Travers et al. 2021), most follow a relatively narrow therapeutic approach and focus (e.g., CBT and motivational interviewing). Given the link between personality disorder and intimate partner violence, there has been some identification that specifically targeting the underlying personality disorder features may be a useful treatment approach (Fruzzetti and Levensky 2000), but this does not seem to be the norm in practice. However, our results highlighting the role of personality functioning in domestic violence and coercive control suggest that empirically validated treatment approaches for personality disorder (e.g., dialectical behaviour therapy, mentalisation‐based therapy and transference‐focused psychotherapy; Leichsenring et al. 2024) may indeed be a fruitful research and clinical avenue for exploration. Second, our results underscore the importance of moving beyond exclusive focus on the particular ‘style’ of expression (i.e., narcissistic, paranoid and histrionic) and instead making efforts to specifically assess for personality functioning in both research and clinical work (as reflected in contemporary dimensional systems, e.g., DSM‐5‐AMPD, ICD‐11 and PDM‐2). Third, these results suggest that when working with severe narcissistic pathology (i.e., ‘malignant’), routine assessment of intimate partner violence and/or coercive control may be necessary, with a focus on this behaviour built into the treatment. Finally, in conducting this research it is also important to highlight the cultural context in which this study takes place, namely, that narcissism is viewed through a strongly stigmatised lens (Penney et al. 2017), particularly on the social Internet (Vorhauer 2024) and news media (Bowen 2019), which can complicate accurate scientific discourse and treatment efforts. As such, a meta‐implication of this study's focus on personality functioning is also the continued effort to reclaim narcissistic pathology within a clinical conceptualisation (Freestone et al. 2020), as opposed to a pejorative one. After all, narcissism is a complex psychiatric condition that often involves immense suffering for both self and others (Crisp and Gabbard 2020; Kealy and Ogrodniczuk 2014) and both requires and deserves psychological treatment.

## 6 Limitations

Some key limitations bear considering as it relates to the findings of this study. First, we have not used comparison groups comprising other personality styles (e.g., dependent and histrionic) in our exploration, and this impacts our ability to draw firm conclusions and specificity of findings. That is, someone with features of profound interpersonal hypersensitivity, passivity, avoidance and unremitting self‐criticism may also be considered to have a severe personality disorder; however, this constellation of impairments in personality functioning may be unlikely to engage in coercive control and violence in the same way reported here. It is our contention that the reported associations between violence and personality functioning may be specific to the context of narcissistic pathology; however, future research should explore this hypothesis. Second, there are demographic imbalances that may limit the generalisability of results. That is, most participants in this study were heterosexual women reporting on a male romantic partner. This gender imbalance is perhaps not unexpected, as narcissistic pathology is typically associated with males (Green et al. 2023), and this is reflected in skewed diagnostic prevalence ratings (males 75% vs. females 25%; American Psychiatric Association 2022). However, authors have highlighted that such gendered division may be a result of cultural stereotype rather than true sex differences (Green et al. 2021) and indeed that females with narcissistic pathology may similarly engage in intimate partner violence (Green et al. 2019; Green et al. 2020). Further, although the majority of participants were reporting on a romantic relationship, there was a subset of participants who were reporting on a family member. Although comparative analyses indicated no differences in relationship type (i.e., family vs. romantic partner) between main variables, there may be significant qualitative differences that are not captured in these data. Indeed, research has highlighted the unique impacts of having a family member with narcissistic pathology (Määttä and Uusiautti 2018) as well as the recognition that children can experience coercive control in domestic violence and abuse contexts also (Callaghan et al. 2018); as such, these are important areas for further research. Finally, the sample size in this study is relatively modest, and as such, the findings would benefit from replication with a larger and more diverse sample, including clinical populations. However, notwithstanding this limitation, as the current sample size was sufficient for the analyses conducted and the population captured (i.e., individuals reporting on close relationships with someone exhibiting pronounced narcissistic features) is relatively unique, this offers valuable insight into a clinically significant phenomenon.

## 7 Conclusion

This study outlines participant endorsement of coercive control and interpersonal violence occurring within relationships (familial or romantic) with relatives with features of pathological narcissism. Although participants rated high levels of both personality disorder and narcissism features in their relative, results indicate that overall impairments in personality functioning show the greatest associations with violence and coercion. These results support contemporary dimensional models of personality disorder and highlight the need to incorporate assessment of personality functioning when working with individuals with narcissistic pathology, as well as the potential value of incorporating personality disorder treatment approaches for interventions targeted towards perpetrators of intimate partner violence and coercive control.

## Author Contributions

Nicholas J. S. Day, David Kealy, Marko Biberdzic, Ava Green and Brin F.S. Grenyer conducted initial conceptualisation, facilitated funding acquisition and developed the research investigation scope and methodology. Nicholas J. S. Day and Georgia Denmeade conducted data curation, formal analysis and visualisation. Nicholas J. S. Day wrote the original manuscript. All authors reviewed the manuscript and contributed to the editing of the final document.

## Ethics Statement

The study received ethical approval from the Institutional Review Board (2023/327) from the University of Wollongong. Participants provided informed consent prior to participating.

## Consent

Participants gave consent for their data to be used for publication.

## Conflicts of Interest

The authors declare no conflicts of interest.

## Data Availability

The datasets generated and/or analysed during the current study do not have clearance to be made available, as participants have not consented for data to be shared outside the research team.
